# Circulating serotonin levels in COPD patients: a pilot study

**DOI:** 10.1186/s12890-018-0730-5

**Published:** 2018-11-08

**Authors:** Pietro Pirina, Elisabetta Zinellu, Panagiotis Paliogiannis, Alessandro G. Fois, Viviana Marras, Salvatore Sotgia, Ciriaco Carru, Angelo Zinellu

**Affiliations:** 1 Department of Respiratory Diseases, University Hospital Sassari (AOU), Sassari, Italy; 2 Department of Clinical and Experimental Medicine, University of Sassari, Sassari, Italy; 3 Department of Biomedical Sciences, University of Sassari, Sassari, Italy

**Keywords:** COPD, Oxidative stress, Serotonin, Markers

## Abstract

**Background:**

Chronic obstructive pulmonary disease (COPD) is a major and increasing global health problem. Serotonin is a neurotransmitter that participates in several pulmonary functions and it has been involved in oxidative stress, which plays essential roles in the pathogenesis of COPD. The current study aimed at establishing the levels of circulating serotonin in COPD, and investigating eventual relations between serotonin and oxidative stress markers.

**Methods:**

Whole blood serotonin was assessed in 43 consecutive patients with stable COPD and in 43 age and sex-matched healthy controls.

**Results:**

Serotonin blood levels were significantly higher in COPD patients than in controls (median 0.81 μmol/L, IQR: 0.61–4.02 vs 0.65 μmol/L, IQR: 0.53–1.39, *p* = 0.02). The univariate logistic regression analysis evidenced that serotonin levels are independently associated with presence of COPD (crude OR = 7.29, 95% CI: 1.296–41.05, *p* = 0.003) and such an association was confirmed also after adjusting for several confounders (OR 21.92, 95% CI 2.02–237.83; *p* = 0.011).

**Conclusions:**

Our study showed higher levels of circulating serotonin in COPD and an inverse correlation with the worsening of airway obstruction. Future studies are necessary to investigate the clinical utility of this finding.

## Background

COPD is an increasing global health problem that nowadays represents the third leading cause of death in the world [1, 2]. It is a chronic progressive disease characterized by a not fully reversible airflow limitation, associated with a chronic inflammation of the lungs and small airways [3, 4]. Although cigarette smoking represents the most known risk factor, in-door and out-door pollution, second-hand smoking and genetic conditions, such as α_1_ antitrypsin deficiency, are considered important additional risk factors [5–7]. The noxious compounds present in cigarette smoke and in environmental pollution, trigger the inflammatory response of the airways and the lungs in susceptible subjects, causing epithelial injury with subsequent production of reactive oxygen species (ROS) [8, 9]. The increased amount of oxidants together with the depletion of antioxidant defenses, results in oxidative stress. It is now recognized that oxidative stress is involved in the pathogenesis of COPD [9, 10] and, in this regard, several biomarkers have been evaluated [11].

Serotonin (5-hydroxytyptamine, 5-HT) is a ubiquitous neurotransmitter that plays important roles in pulmonary functions, being involved in the modulation of respiratory rhythm and in pulmonary vasoconstriction [12, 13]. Furthermore, serotonin has been implicated in the pathogenesis of some of the main comorbidities of COPD, like depression [14, 15] despite the relation between serotonin, COPD and depression is still to be verified.

Moreover, serotonin has been reported to induce oxidative stress via monoamine oxidase-dependent pathway in human heart valves [16] and in mesenchymal stem cells [17] indicating that serotonin metabolism may be involved in oxidative stress. Its role in the genesis and maintenance of oxidative stress in COPD patients is not well-established. In this pilot study we focused on the assessment of blood serotonin levels in COPD patients compared to healthy controls and in relation to airway obstruction severity; we have also evaluated potential associations between serotonin and oxidative stress markers, like thiobarbituric acid reactive substances (TBARS) and protein sulfhydryl groups (PSH), which have been demonstrated in previous studies to be altered in patients with COPD [18, 19].

## Methods

### Subjects

This case–control pilot study involved 43 consecutive patients with stable COPD (mean age 74.8 ± 5.9 years, range 52–85 years), treated at the Respiratory Unit of the University of Sassari.

The diagnosis of COPD was made in accordance with the Global Initiative for Chronic Obstructive Lung Disease criteria [20]. The patients enrolled did not have a previous diagnosis of COPD and they were not under treatment with long- or short-acting β-agonists, or long-acting muscarinic antagonists, as well as with inhaled corticosteroids at least within 4 weeks prior to enrollment. Each patient performed respiratory function tests and underwent physical examination, blood tests and chest radiographs. The functional diagnosis of COPD was based on the presence of not fully reversible airflow limitation, defined by a post-bronchodilator ratio of forced expiratory volume in 1 s to forced vital capacity (FEV1/FVC) < 70% of the predicted value [4]. In order to collect demographic and clinical data, including smoking history and information about occupational and/or in-door and out-door pollutants exposure, a structured questionnaire was administered. In particular, patients who were never smokers, had been exposed to other COPD risk factors: half of them were women exposed to second-hand smoke, three of them had worked as miners and therefore exposed to silica powders and two of them had been exposed to indoor pollutants (biomass heating, etc).

A group of 43 age- and sex-matched healthy controls, with no medical history, was also included. Subjects with severe concomitant diseases, such as heart diseases, kidney and liver diseases, systemic inflammatory diseases, patients with severe COPD and patients with a history of asthma and atopic diseases, were excluded from the study. The study was approved by the Institutional Local Ethics Committee (Azienda Sanitaria Locale n°1 di Sassari (Italy) (prot. 2175/CE del 21/04/2015). The subjects who decided to participate, signed a written informed consent before enrollment.

### Biochemical analysis

The levels of serotonin in whole blood of COPD subjects and healthy controls were determined according to a method previously described [21]. The inter-assay CV was < 8%. The oxidative stress indices TBARS and PSH were measured as previously reported [22, 23]. TBARS assay was employed to measure malondialdehyde (MDA) and other aldehydes produced by lipid peroxidation induced by reactive oxygen species. TBARS were determined by measuring the absorbance at 535 nm after reaction with thiobarbituric acid. A calibration curve was obtained using MDA as reference standard. Plasma PSH determination was performed by spectrophotometry with 5,5′-dithiobis-2-nitrobenzoic acid (DTNB) as titrating agent by measuring the absorbance of conjugate at 405 nm. Concentration in samples was determined from a GSH standard curve. ROS can oxidize protein SH to disulfide or sulfenic acid, leading to a reduction in –SH groups.

### Statistical analysis

The results are expressed as mean (mean ± SD) or median values (median and IQR). The distribution of variables was evaluated by means of Shapiro-Wilk test. The statistical comparisons between groups were assessed by means of unpaired Student’s t-test or Mann-Whitney rank sum test, as appropriate. Correlations between variables were estimated using Spearman’s or Pearson’s correlation, as appropriate. In order to verify the presence of association between variables potentially implicated in disease development, logistic regression analysis was performed. Receiver operating characteristics (ROC) curve analysis was used to test the ability of serotonin to predict COPD, alone and in combination with TBARS and PSH. ROC curves were obtained with calculation of the area under the curve (AUC). Optimal cut-off maximizing sensitivity and specificity was selected according to the Youden Index.

Statistical analyses were performed using MedCalc for Windows, version 15.4 64 bit (MedCalc Software, Ostend, Belgium) and SPSS for Windows, version 14.0 32 bit (IBM Corporation; Armonk, NY, USA).

## Results

**Fig. 1:**
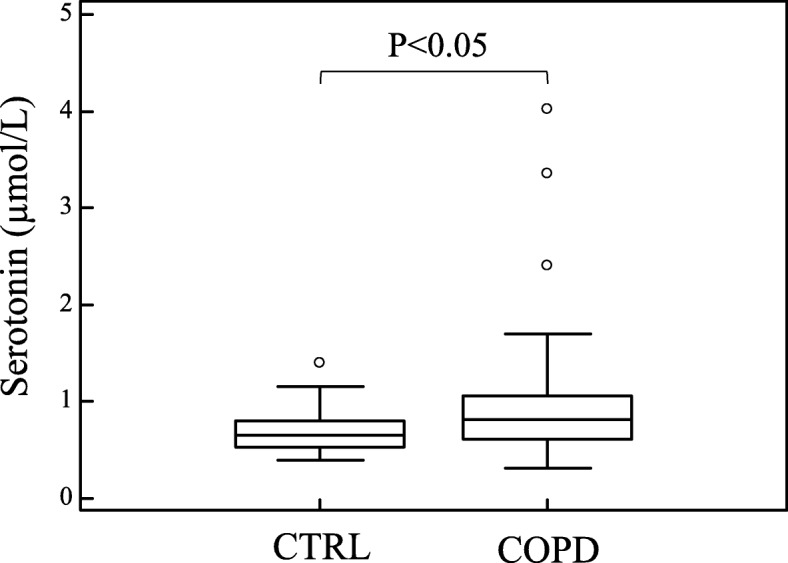
Blood levels of serotonin in healthy subjects (*n* = 43) and in the totality of COPD patients (*n* = 43). The central horizontal line on each box represents the median, the ends of the boxes are 25 and 75 percentiles and the error bars 5 and 95%. *P*-values derived from Student’s t-test

**Fig. 2:**
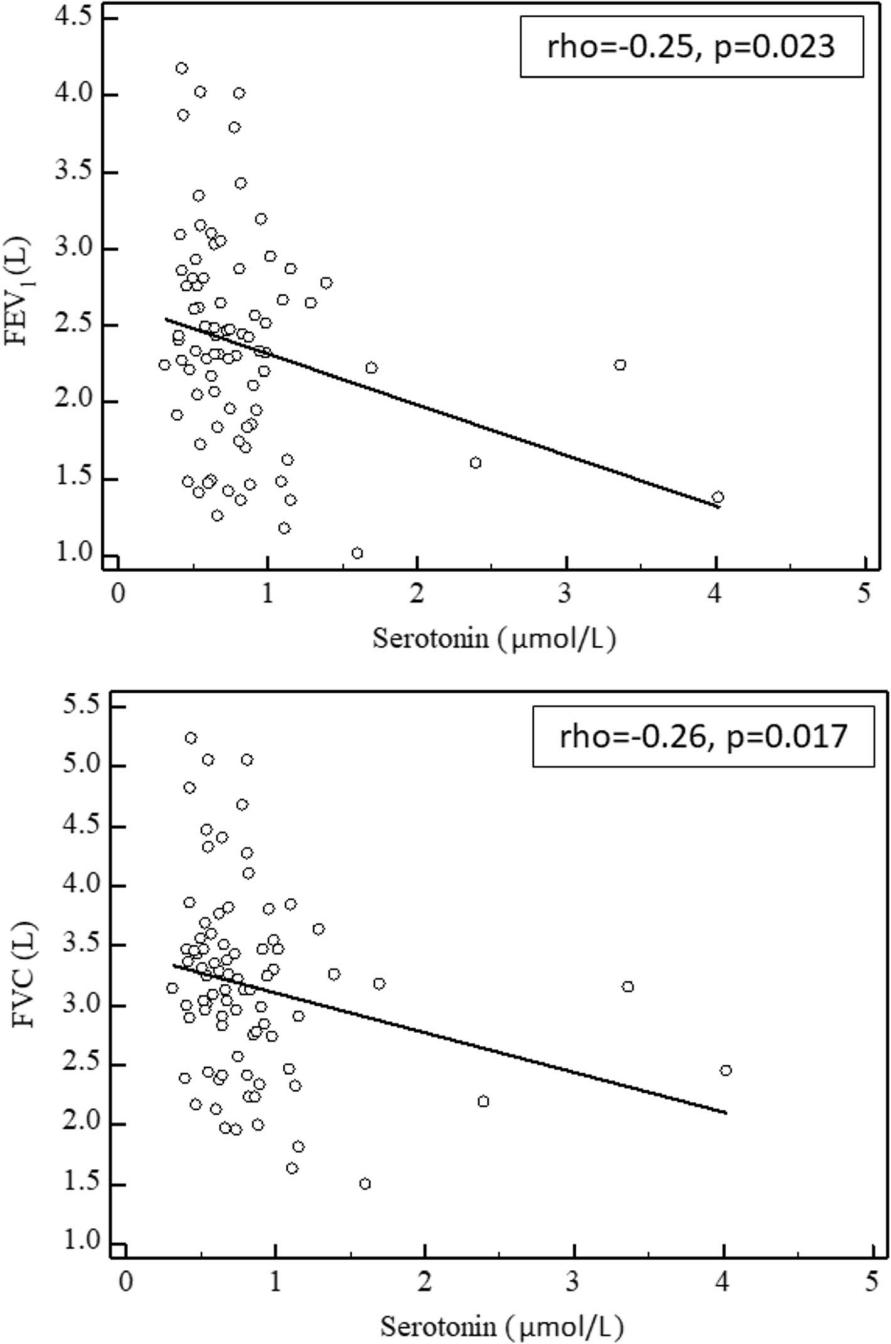
Correlation between FEV1 and FVC with blood serotonin in COPD patients

Table 1 reports the demographic and clinical characteristics in controls and COPD patients. As expected, COPD patients showed a reduced FEV_1_ and FEV_1_/FVC ratio. There were no between-group differences in smoking status or BMI. Serotonin blood levels were significantly higher in COPD patients (median 0.81 μmol/L, IQR: 0.61–4.02) than in controls (median 0.65 μmol/L, IQR: 0.53–1.39), *p* = 0.02 (Fig. 1). As previously reported TBARS concentrations significantly increased, and PSH concentrations significantly decreased, according to COPD presence [18]. However, no significant correlations were observed between serotonin blood levels and oxidative stress indices. As reported in Fig. 2, Spearman’s correlations in the whole study population indicated that serotonin blood values are inversely associated with FEV_1_ (rho = − 0.25, *p* = 0.023) and FVC (rho = − 0.26, *p* = 0.017). Table 2 summarizes the results of the univariate logistic regression analysis, which evidenced that serotonin levels were independently associated with presence of COPD (crude OR = 7.29, 95% CI: 1.296–41.05, *p* = 0.003). This association remained significant also after adjusting for age, gender, BMI, smoking status, and oxidative stress indices (OR 21.92, 95% CI 2.02–237.83; *p* = 0.011).

**Table 1:** Clinical and functional parameters of healthy subjects and COPD patients

	Controls (*n* = 43)	COPD (*n* = 43)
Age (years)	73 ± 7	75 ± 6
Gender (F/M)	9/34	9/34
BMI (kg/m^2^)	26 ± 4	27 ± 4
Never smoked	14 (33%)	10 (23%)
Current smokers	3 (7%)	3 (7%)
Ex smokers	26 (60)	30 (70%)
FEV1 (L)	2.8 ± 0.6	2.0 ± 0.6**
FEV1 (% predicted)	112 ± 14	80 ± 18**
FVC (L)	3.4 ± 0.7	2.9 ± 0.8*
FVC (% predicted)	108 ± 15	88 ± 15**
FEV1/FVC	80.4 ± 3.9	66.6 ± 4.8**
RV (L)	2.0 ± 0.5	3.4 ± 0.9**
RV (% predicted)	105 ± 12	137 ± 32**
TLC (L)	6.0 ± 1.1	6.4 ± 1.1
TLC (% predicted)	107 ± 10	108 ± 14
RV/TLC (%)	32 ± 3	53 ± 9**

*FEV1* Forced Expiratory Volume in the 1st second, *FVC* Forced Vital Capacity, *FEV1/FVC* Tiffeneau index (calculated as LLN)

*p < 0.01, **p < 0.001 obtained by Student’s t-test

**Table 2:** Predicting factors for chronic obstructive pulmonary disease

Factor	Univariate analysis			Multivariate analysis		
	Crude OR	95%CI	*p*-value	OR	95%CI	*p*-value
Age	1.035	0.967–1.109	0.749	0.998	0.917–1.088	0.978
Gender	1.000	0.368–2.720	0.061	1.567	0.341–7.203	0.563
BMI	1.076	0.953–1.216	0.692	1.108	0.942–1.303	0.217
Smoking status	1.404	0.551–3.581	1.000	0.516	0.144–1.854	0.319
PSH	0.427	0.258–0.705	0.0001	0.289	0.144–0.580	0.0005
TBARS	1.816	1.053–3.131	0.02	2.947	1.322–6.570	0.008
Serotonin	7.294	1.296–41.05	0.003	21.92	2.02–237.83	0.011

*BMI* body mass index, *PSH* protein sulfhydryl groups, *TBARS* thiobarbituric acid reactive substances, *OR* odds ratio, *CI* confidence interval

**Fig. 3:**
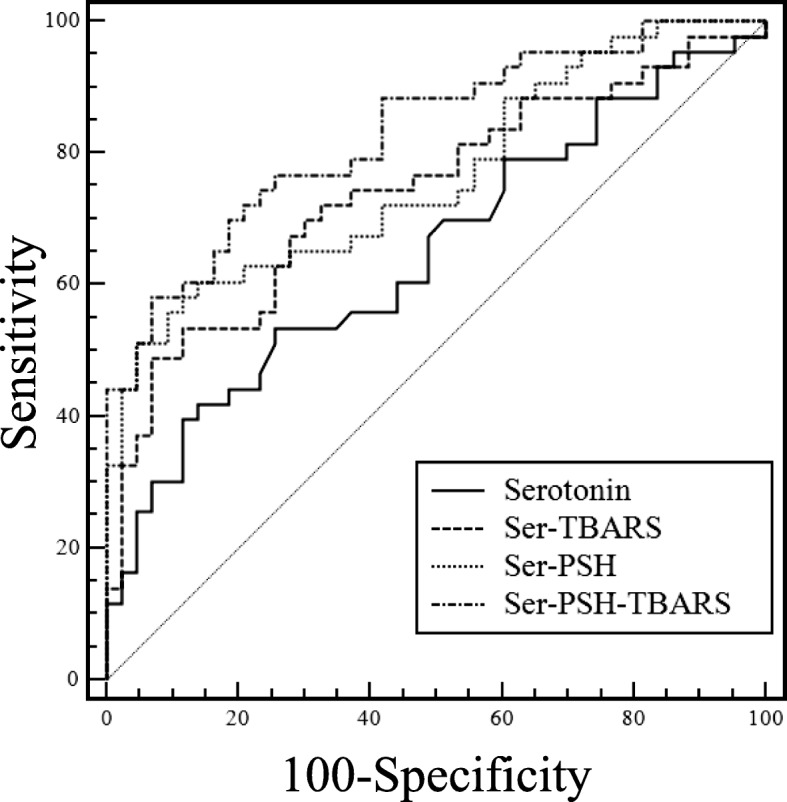
The area under receiver operating characteristic curves of serotonin

ROC curve analysis was performed to evaluate the sensitivity, specificity, and diagnostic accuracy of serum serotonin levels alone, or in combination with PSH and TBARS, in distinguishing COPD from healthy subjects (Fig. 3 and Table 3). Serotonin alone, with a cut-off of 0.78 μmol/L discriminated COPD from controls with 53.5% sensitivity and 74.4% specificity (AUC = 0.647, 95% CI 0.537–0.747, *p* = 0.014). Serotonin in combination with PSH and TBARS produced the best result, with an AUC of 0.830 (95% CI 0.733–0.902, *p* < 0.0001), sensitivity 76.7% and specificity 74.4%. Pairwise comparison of ROC curves indicated that the combination of serotonin, PSH and TBARS yield a significant increase in AUC (+ 0.183, *p* = 0.0035) compared to AUC obtained with serotonin alone.

**Table 3:** Prognostic accuracy of serotonin alone or in combination with TBARS and MDA

Marker	AUC	95%CI	*p* value	Cut-off	Sensib	Specif
Serotonin	0.647	0.537–0.747	0.014	> 0.780	53.5%	74.4%
Ser-TBARS	0.741	0.635–0.829	< 0.0001	> 0.543	53.5%	88.4%
Ser-PSH	0.764	0.660–0.849	< 0.0001	> 0.569	60.5%	86.1%
Ser-PSH-TBARS	0.830	0.733–0.902	< 0.0001	> 0.460	76.7%	74.4%

*PSH* protein sulfhydryl groups, *TBARS* thiobarbituric acid reactive substances, *MDA* malondialdehyde, *AUC* area under the curve, *CI* confidence interval

## Discussion

Serotonin is a biogenic amine known for its role as a neurotransmitter. It is synthesized from L-tryptophan within the central nervous system (CNS), where it is stored in the presynaptic neurons. Serotonin synthesis outside the CNS is limited to enterochromaffin cells, while platelets take up serotonin from plasma representing a further major storing site for serotonin [24]. The main metabolic pathway of serotonin is the metabolism by monoamine oxidase (MAO) that catalyses the oxidative deamination of the amine substrate, with production of its aldehyde intermediate and hydrogen peroxide as a by-product. The aldehyde intermediate is then rapidly oxidized by aldehyde dehydrogenase to 5-hydroxyindoleacetic acid [24, 25].

It is known that lung represent an important site in which removal and metabolism of serotonin take place [26]. The ability of the endothelial cells of the lungs to metabolise amines may be reduced in disease states, and this could explain their increased levels in the circulation. Elevated circulating levels of serotonin have been reported in respiratory diseases such as asthma [27] and lung cancer [28]. COPD has also been associated with a variation of a transporter gene involved in serotonin re-uptake [29] and metabolites of the serotonin pathway have been associated with adverse outcome in exacerbated COPD [30]. Furthermore, it is now recognized that oxidative stress is involved in the pathogenesis of COPD [9, 10] and it has been reported that serotonin induces oxidative stress via MAO-dependent pathway in human heart valves [16] and in mesenchymal stem cells [17]. Moreover, it has been described that cigarette smoke, a major COPD risk factor, inhibit MAO in different species in vitro [31]. Such evidences suggest that serotonin may play relevant roles in the pathogenesis of COPD.

Our study has evidenced a significant increase in serotonin levels in COPD patients compared to controls. Spearman’s correlations indicated that blood serotonin values are inversely associated with FEV1 and FVC, to confirm an association of serotonin levels not only with the presence of COPD, but also with the severity of airway obstruction. The univariate logistic regression analysis has shown that serotonin levels were independently associated with presence of COPD also after adjusting for age, gender, BMI, smoking status, and oxidative stress indices.

Lau et al. [32], investigated the role of serotonin in the pathogenesis of COPD and found higher levels of circulating serotonin in patients compared to healthy controls. Unlike us, they examined only male COPD subjects who were significantly older than controls, and found a positive correlation between serotonin levels and age in pathological subjects. Moreover, in their analysis they prevalently included moderate to very severe COPD cases, finding no differences in serotonin levels according to disease progression. In our study, we confirmed the presence of higher blood serotonin levels in COPD compared to age- and sex-matched controls. As opposed to the study of Lau et al., our patients had a mild-moderate degree of airway obstruction (FEV1 > 50%). From this point of view these patients can be considered in the early phase of the disease. In fact, they were all newly diagnosed patients who had not yet started a treatment. These data support the hypothesis that serotonin could be a predictive marker of the onset of COPD. Moreover, the inverse correlation that we found between serotonin levels and FEV1 and FVC, suggests a relation of this molecule with the worsening of airway obstruction.

The ROC curve analysis for serotonin significantly discriminate patients with COPD from those without COPD and showed that the diagnostic accuracy is higher when serotonin is combined with TBARS and PSH. In particular, the triple combination of serotonin, TBARS and PSH increased significantly the AUC of the ROC curve. Although it is an interesting result, its clinical validity and usefulness needs to be further investigated. Moreover, Spearman’s correlation analysis failed to find a relationship between serotonin and oxidative stress biomarkers. This could be due to the low number of subjects involved, in particular to the absence of severe COPD subjects. The small number of cases, and the lack of advanced stage COPD patients represent the main limitations of our work. On the other hand, our study is the first to investigate blood serotonin levels in a cohort of early COPD cases and has several strengths, like its prospective case-match design, the accurate statistical analysis, and the research of associations with other well-established biomarkers of oxidative stress.

## Conclusions

Our study confirms literature data showing an involvement of serotonin in the pathogenesis of COPD, demonstrating a statistically significant increase of circulating serotonin levels in an early phase of the disease, and a relation with the worsening of the airway obstruction. Given the need of biomarkers useful to detect and monitor COPD and its response to treatments, this seems to be a promising result that need to be further investigated.

## Abbreviations

5-HT: Serotonin
AUC: Area under the curve
BMI: Body mass index
CNS: Central nervous system
COPD: Chronic obstructive pulmonary disease
FEV1: Forced expiratory volume in the 1st second
FVC: Forced vital capacity
MAO: Monoamine oxidase
MDA: Malondialdehyde
PSH: Protein sulfhydryl groups
ROC: Receiver operating characteristics
TBARS: Thiobarbituric acid reactive substances
